# Correction: Magnetoconductance modulations due to interlayer tunneling in radial superlattices

**DOI:** 10.1039/d2nh90054h

**Published:** 2023-01-05

**Authors:** Yu-Jie Zhong, Angus Huang, Hui Liu, Xuan-Fu Huang, Horng-Tay Jeng, Jhih-Shih You, Carmine Ortix, Ching-Hao Chang

**Affiliations:** a Department of Physics, National Cheng Kung University Tainan 70101 Taiwan cutygo@phys.ncku.edu.tw; b Center for Quantum Frontiers of Research & Technology (QFort), National Cheng Kung University Tainan 70101 Taiwan; c Department of Physics, National Tsing Hua University Hsinchu 30013 Taiwan; d Center for Quantum Technology, National Tsing Hua University Hsinchu 30013 Taiwan; e IFW Dresden and Würzburg-Dresden Cluster of Excellence ct.qmat Helmholtzstrasse 20 01069 Dresden Germany; f Institute of Physics, Academia Sinica Taipei 11529 Taiwan; g Department of Physics, National Taiwan Normal University Taipei 11677 Taiwan; h Institute for Theoretical Physics, Center for Extreme Matter and Emergent Phenomena, Utrecht University Princetonplein 5 NL-3584 CC Utrecht The Netherlands; i Dipartimento di Fisica “E. R. Caianiello”, Universitá di Salerno IT-84084 Fisciano Italy

## Abstract

Correction for ‘Magnetoconductance modulations due to interlayer tunneling in radial superlattices’ by Yu-Jie Zhong *et al.*, *Nanoscale Horiz.*, 2022, **7**, 168–173, https://doi.org/10.1039/D1NH00449B.

The authors regret an error in the final line of eqn (3) of the published article. The corrected form of eqn (3) is shown here:3
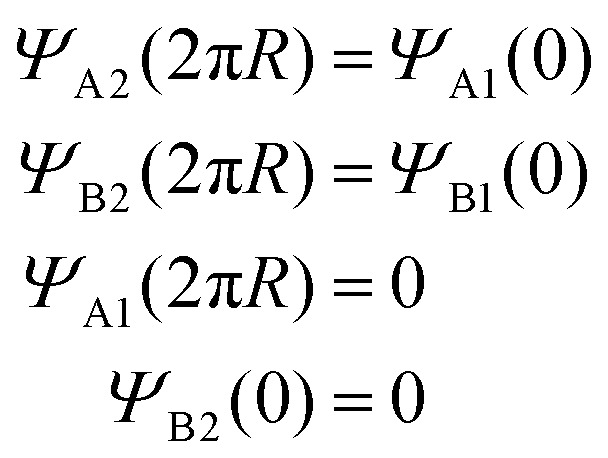


The Royal Society of Chemistry apologises for these errors and any consequent inconvenience to authors and readers.

## Supplementary Material

